# Detecting the Collapse of Cooperation in Evolving Networks

**DOI:** 10.1038/srep30845

**Published:** 2016-08-05

**Authors:** Matteo Cavaliere, Guoli Yang, Vincent Danos, Vasilis Dakos

**Affiliations:** 1 School of Informatics, University of Edinburgh, Scotland, United Kingdom; 2 CNRS-ENS, Paris, France; 3 Institute of Integrative Biology, Center for Adaptation to a Changing Environment, ETHZ, Zürich, Switzerland

## Abstract

The sustainability of biological, social, economic and ecological communities is often determined by the outcome of social conflicts between cooperative and selfish individuals (cheaters). Cheaters avoid the cost of contributing to the community and can occasionally spread in the population leading to the complete collapse of cooperation. Although such collapse often unfolds unexpectedly, it is unclear whether one can detect the risk of cheater’s invasions and loss of cooperation in an evolving community. Here, we combine dynamical networks and evolutionary game theory to study the abrupt loss of cooperation with tools for studying critical transitions. We estimate the risk of cooperation collapse following the introduction of a single cheater under gradually changing conditions. We observe an increase in the average time it takes for cheaters to be eliminated from the community as the risk of collapse increases. We argue that such slow system response resembles slowing down in recovery rates prior to a critical transition. In addition, we show how changes in community structure reflect the risk of cooperation collapse. We find that these changes strongly depend on the mechanism that governs how cheaters evolve in the community. Our results highlight novel directions for detecting abrupt transitions in evolving networks.

The sustainability of many biological, social, economic, and ecological communities is determined by the interplay between individual actions and collective dynamics^1^. The successful performance of a community is often based on the cooperative attitude of individuals that pay a personal cost to distribute general benefits^2^. Nonetheless, although cooperation favors in general the success of a community, it can also facilitate the appearance of *cheaters* who take advantage of *cooperators*, spread in the community, and may even cause its collapse^1^. The failure of cooperation in the presence of cheaters has been observed in many systems at different scales. For instance, cooperating cells of *Pseudomonas fluorescens* build biofilms that help them to grow better, while mutant cells (cheaters) - that do not produce similar adhesive factors - take advantage of the existing structure in order to spread and to eventually cause the colony to collapse^3^,^4^. Fruiting bodies formed under starvation by cooperative cells of *Myxococcus xanthus* can also be invaded by cheaters leading to the disruption of the fruiting body structure and forcing the cooperative survivors to reinvest in reconstruction^5^. At a different scale and in a different context shifts in cooperation and cheating have been debated to be causes of economic crises^6^, as well as causes for the “tragedy” of common pool resources, (e.g., from fisheries to forests)^7^,^8^.

In all these systems a long-standing question has been to understand the mechanisms that allow cooperators to resist the reproductive advantage of selfish cheating individuals^2^. Among the many theoretical and experimental studies on the maintenance of cooperation^2^,^9^,^10^,^11^, scenarios where strategies co-evolve with population structure^12^ are of particular interest as they show how structural properties in the population can affect the evolution of cooperation^10^,^11^,^12^,^13^. For instance, it has been shown that not only the number of cheaters in the community is important, but also how and to whom cheaters are connected^14^, as well as the mechanisms employed by the players to choose their interaction partners^15^,^16^,^17^. The interplay between the way individuals are connected and the overall prosperity of a system^1^,^18^ endogenously determines either the formation or the sudden collapse of cooperative communities^1^. Despite our relative good understanding of the conditions that promote the failure of cooperation in a community, it is still difficult to predict whether the appearance of a cheater will cause the eventual loss of cooperation^7^,^8^. This is because it is hard to identify the underlying conditions that increase the risk of collapse in practice. Thus, it is crucial to develop alternative ways for detecting the risk of collapse of cooperation in a structurally evolving community.

Recent work has suggested that generic patterns in the dynamics of a system can be used to infer proximity to abrupt and unexpected changes termed critical transitions^19^. These dynamical patterns are generic in the sense that they do not depend on the particular system in question, but they are determined by the mathematical phenomenon of critical slowing down (CSD) that occurs prior to local bifurcation points^20^,^21^. A bifurcation point represents a threshold where a qualitative change in the equilibrium of a system takes place: the iconic case is the shift between two alternative equilibria at a crossing of a fold bifurcation. Close to a local bifurcation, CSD implies that the system takes longer to recover back to equilibrium after a disturbance^19^,^22^. In addition, the dynamics of the system become strongly variable^23^, and highly autocorrelated^24^. As such, decreasing recovery rate, rising variance, and rising autocorrelation can all be used as early-warning signals of approaching critical transitions^19^,^25^, or more general as indicators of loss of resilience^26^. CSD indicators have been identified in a variety of systems: from the collapse of cyanobacteria^27^, zooplankton^28^, and yeast populations^29^ in the lab, to changes in trophic structure in lakes^23^, as well as prior to abrupt past climatic events^25^, and the onset of depression in human patients^30^. Despite limitations and challenges in detecting resilience indicators^31^,^32^, growing evidence supports their potential use across different disciplines^33^. The application of such indicators for detecting abrupt transitions in structurally complex communities is still, however, largely unexplored. There are few studies that have highlighted the emergence of tipping points in mutualistic communities of plants and their pollinators^34^, and the detection of transitions in ecological^26^ and socio-ecological networks^35^. Nonetheless, these studies assume a static structure that does not allow changes in the interactions among network components.

Here, for the first time to our knowledge, we combine evolutionary game theory and dynamical networks to detect the collapse of cooperation in an evolving (structurally dynamic) community, using resilience indicators for critical transitions. To do this, we adapt a network model that displays cooperation collapses as consequence of cheater’s invasions^14^. Evolution of cheaters and cooperators is based on social inheritance^36^: newcomers copy strategies and connections of successful role-models. Increased ability of newcomers to link to a high number of individuals already present in the network allows high prosperity but increases the risk of facilitating cheater’s invasions that can lead to the collapse of cooperation^14^. We show that the collapse of cooperation occurs in a rather abrupt non-linear way. Prior to collapse we estimate a series of indicators (structural and non-structural) that can signal the increasing fragility of cooperation. Our contribution is twofold. First, we argue how the loss of cooperation in evolving communities may resemble features of a critical transition. Second, we develop a set of structural-based indicators and state-based indicators for detecting approaching transitions in structurally evolving communities.

## Results

### Cooperation in a Dynamical Network

We consider a network model as introduced by^14^. The model consists of a fixed number of agents (nodes) but with a non-fixed number of links: to whom and to how many neighbours an agent is connected varies during the evolution of the system. Each agent in the network adopts one of the two strategies of the *Prisoner’s Dilemma*. A *cooperator* pays a cost *c* to provide a benefit *b* to all of its neighbours; a *cheater* pays no cost and distributes no benefit. This translates into a game with payoff matrix:


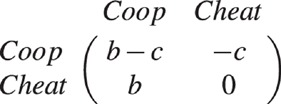


**Figure 1:**
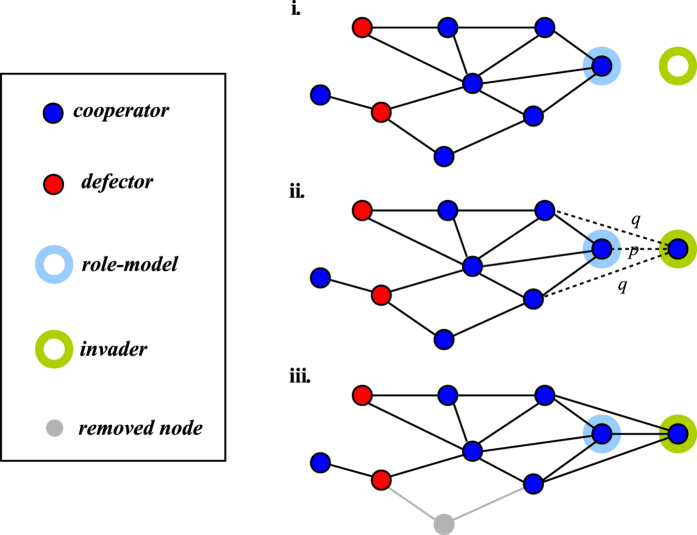
Evolution in a dynamical network. The dynamics follow three distinct update steps in the considered model: (step i) A role-model is selected based on its effective payoff. (step ii) The invader (newcomer) connects to the role-model with a probability *p* (dashed line), connects to each of its neighbours with a probability *q* (dotted lines) and emulates its strategy. *p* and *q* are called embedding parameters. (step iii) A randomly selected node and all its connections are removed from the network.

For instance, if a cooperator has *m* cooperative neighbours and *n* cheating neighbours, its payoff is *m*(*b* − *c*) − *nc*. A cheater in the same neighbourhood has payoff *mb*. We use *b*/*c* = 3 in our numerical experiments. The dynamics of the system are defined by a discrete sequence of *update steps*(Fig. 1). At each update step a new node (a *newcomer*) is added and a randomly chosen existing node is removed from the system so that the number of nodes is constant (*N* = 100). As the newcomer has no specific strategy, a node *i* is selected as a role-model with a probability proportional to its *effective payoff EP*_*i*_ = 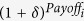, where *δ* ≥ 0 specifies the intensity of selection and *Payoff*_*i*_ is the sum of pair-wise interactions of each node. For *δ* = 0 the selection probability is same for all nodes, while increasing *δ* makes it more likely that a newcomer chooses a node with a higher payoff. A newcomer adopts the strategy of the role-model and connects with it with a probability *p* as well as with each of its neighbours with a probability *q* (that means *q*^*k*^ is the probability that a newcomer connects to all *k* neighbours of the role-model). The parameters *p* and *q* are called *embedding parameters*. They explicitly determine the ability of the newcomer to imitate the role-model’s social network. In particular, the embedding parameter *q* determines the ability of newcomers to link to a high number of agents in the network, while parameter *p* dictates the probability that the newcomer connects to its role model (we fix *p* = 0.6, other values of *p* are considered in the Supplement). The degree of competition, *selection strength*, is controlled by parameter *δ*, which determines the effective payoff. We assume that newcomers always copy the strategy of the selected role-model and there is no possibility for a newcomer to change its strategy by chance; in other words we neglect the possibility of mutations. Following these rules, the structure of our communities is an emergent property that is determined by the ability of a newcomer to select a role-model and mimic its structure based on the fitness of all nodes in the network. This is different from classical models of evolutionary game theory where the community structure is fixed and nodes change their strategy depending only on the strategy of their neighbours^37^.

### The Collapse of Cooperation

We studied how increasing *q* slowly erodes the stability of cooperation and increases the risk of cooperation collapse by performing a series of perturbation experiments. A perturbation experiment is defined as the introduction of an invader (e.g. cheater) in a network where all agents have the opposite strategy (e.g. cooperators).

**Figure 2:**
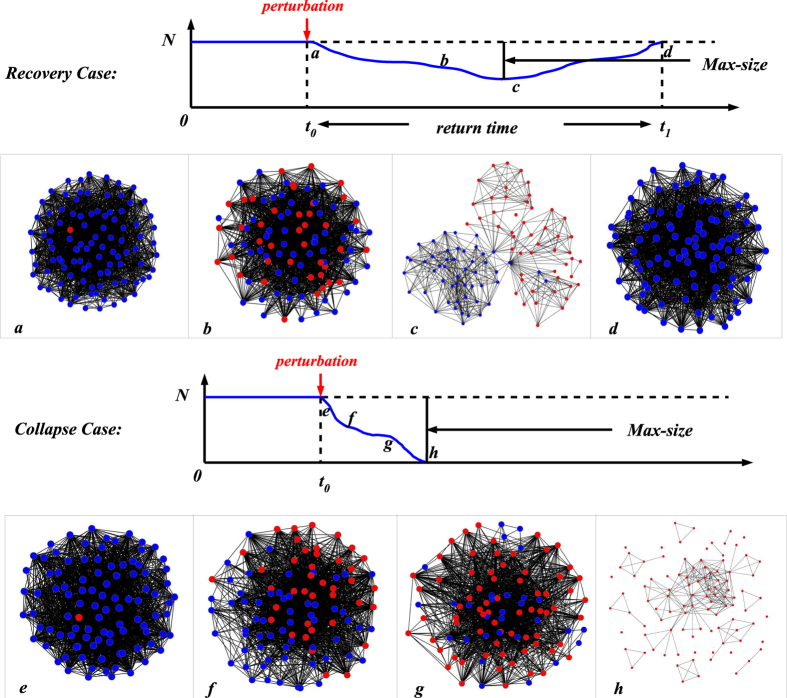
Recovery and Collapse of Cooperation in Perturbation Experiments. Recovery and collapse of cooperation following the addition of a single invader in a network with agents of the opposite strategy. We show the two possible outcomes of a perturbation obtained by adding a cheater in a network of all cooperators. The first row shows the typical stages (and network topologies) of an unsuccessful perturbation where cooperators resist, while the bottom row shows the typical stages (and network topologies) of a successful perturbation where cheaters invade (in both cases *q* = 0.8). Note the distinct network structure in community of all cooperators (highly connected) and in a community of all cheaters (highly fragmented).

**Figure 3:**
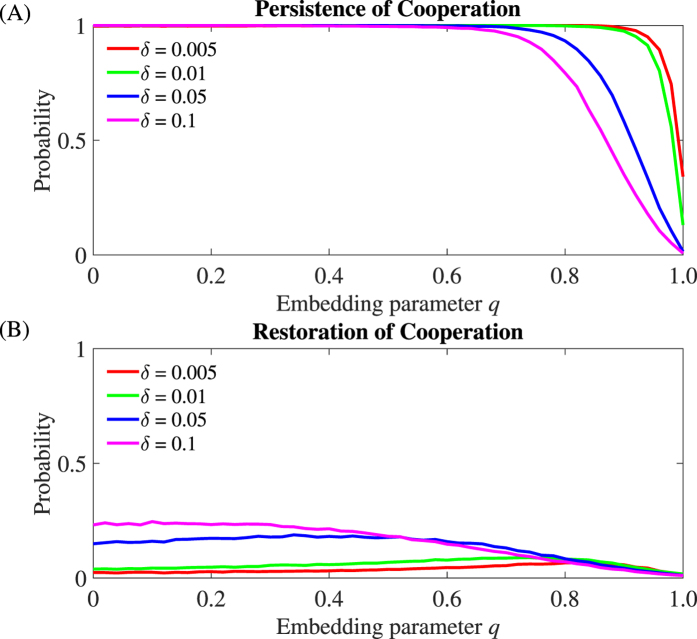
Persistence and Restoration of Cooperation. (**A**) Persistence of cooperation as a function of the embedding parameter *q* for various selection strengths *δ*. Note the non-monotonicity of the curves (visible clearly in the inset panel): for a low selection *δ*, the persistence of cooperation reaches a maximum before collapsing. As selection becomes stronger, the persistence of cooperation decreases monotonically. (**B**) Restoration of cooperation as a function of the embedding parameter *q* for various selection strengths *δ*. Persistence probabilities are computed as 1 − *ψ* and restoration probabilities are computed as *ψ*, where *ψ* is the fraction of perturbations that lead to the successful invasion of the mutant out of 20.000 perturbation experiments. Each perturbation is done by updating the network of (**A**) all cooperators or (**B**) all cheaters for a long time following the addition of a mutant (all results are shown at *p* = 0.6; see Supplementary Material Figure S2 for different *p*s).

We consider perturbations in a network of all cooperators after we have iterated the model to establish a quasi-stable network topology. We then introduce a cheater and we monitor the system until the cheater either fails to invade, *recovery*, or the cheater invades and cooperation collapses, *collapse* (Fig. 2). Using this approach, we evaluate the fraction of perturbations that lead to the collapse of cooperation for increasing values of the embedding parameter *q* that controls the links established between the invader and the rest of the network. The fraction of perturbations that lead to cooperation collapse represents the probability *ψ* of an invading cheater to spread and trigger the collapse of cooperation. We call the complement of this probability *cooperation persistence* (1 − *ψ*). We find that cooperation is uninvadable for a wide range of the control parameter *q*. Only when *q* crosses a certain threshold, cooperation is doomed to fail and its loss occurs in an abrupt non-linear but continuous way (Fig. 3A). The exact value of the threshold depends on the selection strength *δ*. A high *δ* causes an earlier collapse and increases the probability that a single cheater invades successfully in the community (Fig. 3A). On the other hand, parameter *p* has only a slight effect on the resilience of cooperation, as it just affects the probability of the single link between the invader and the role-model (Figure S2 in the Supplementary Material).

Once cooperation collapses, its restoration by re-introducing a cooperator in a network of cheaters is difficult. In this reverse scenario, we find that although the probability of a single cooperator to invade a network of cheaters increases for decreasing embedding parameter *q*, it never reaches 1 (Fig. 3B). In other words, once cooperation is lost it is difficult to be restored. This indicates that cheaters’ dominance is much more resilient than cooperators’ dominance. Restoring cooperation is only possible by inducing a stronger perturbation: an invasion of more than a single cooperator (Figure S1 in the Supplementary Material).

### Structural and Non-structural Indicators for Detecting the Collapse of Cooperation

The abrupt loss of cooperation raises the question whether it is possible to detect it in advance. Measuring the strength of parameter *q* is not only difficult in practice, but also largely uninformative as for a range of low *q*’s there is little sign of change in the persistence of cooperation (Fig. 3). Thus, alternative diagnostic tools would be desirable in order to estimate the rising risk of cooperation collapse. Motivated by the possibility to detect upcoming critical transition using generic indicators of resilience^19^, we explore whether we can use similar indicators as precursors for the eroding stability of cooperation.

**Figure 4:**
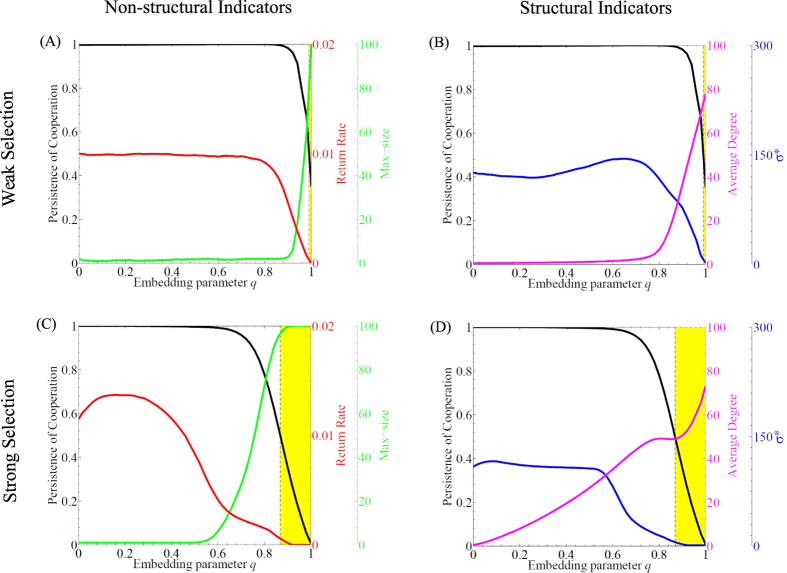
Detecting the Loss of Cooperation. Structural and non-structural indicators for detecting the loss of cooperation after the invasion of a single cheater at increasing levels of embedding parameter *q*. Non-structural indicators are return rate (inverse of the number of update steps the system takes to go back to its original state following a perturbation), and max-size (the maximal number of cheaters recorded during a perturbation). Structural indicators are structural coefficient σ^*^ that evaluates the ratio between the cooperative interactions and the exploitative interactions, and average degree (the average number of links per node). Upper row: weak selection, *δ* = 0.005; lower row: strong selection, *δ* = 0.1. The black curves denote cooperation persistence. The yellow shaded area identifies the values of *q* where cooperation persistence falls below 0.5. Each point of the indicators is the median estimated out of 20.000 perturbation experiments.

In particular, we evaluate two broad classes of indicators: those based on the dynamics of community composition (that we call *non-structural*) and those based on community structure (that we call *structural*). The difference between the two classes is that non-structural indicators reflect changes in the numbers of cheaters and cooperators (demographic changes), whereas structural indicators reflect changes in the interactions between cheaters and cooperators (topological changes). We estimate two non-structural indicators: a) return rate, the inverse of the time it takes for the system to recover back to its original state of full cooperation after the addition of a single cheater (recall that time is measured in update steps in our model), and b) maximum size, the maximal amount of cheaters possible to spread after the introduction of a single cheater. We also focus on two structural metrics: a) average degree (or connectance), the average number of links of each agent in the network, and b) the relative amount of cooperative interactions σ^*^, that is the fraction of beneficial interactions between cooperators over the exploitative interactions between cooperators and cheaters (see Methods for details). We also tested for other structural indicators that we present in the supplement (Section 7). We estimate all indicators for each perturbation experiment at increasing values of the embedding parameter *q* and for different selection strengths *δ*. In Fig. 4, we plot the median values from 20.000 perturbations performed at each *q* (Figure S6 summarizes the distributions from all indicators). We find that both non-structural and structural indicators change in distinct ways before cooperation collapses. In particular, we compare trends among indicators up to the value of *q* at which the probability of cooperation persistence drops below 0.5. We use this threshold value as the most conservative choice for the onset of cooperation collapse. We observe the strongest changes for all indicators close to the transition except for average degree when selection strength *δ* is high (Fig. 4D). In general, strong selection (*δ* = 0.1) leads to more pronounced and earlier changes in the trends when compared to weak selection (Figure S3 summarizes different selection strengths). Interestingly, return rate also decreases before cooperation collapses (Fig. 4A,C). As decreasing return rates are signatures of critical slowing down prior to local bifurcation points^22^, this finding indicates that the loss of cooperation in evolving networks might bear similar dynamical features to a critical transition.

### Assessing the Consistency and Early Detection of the Indicators

**Figure 5:**
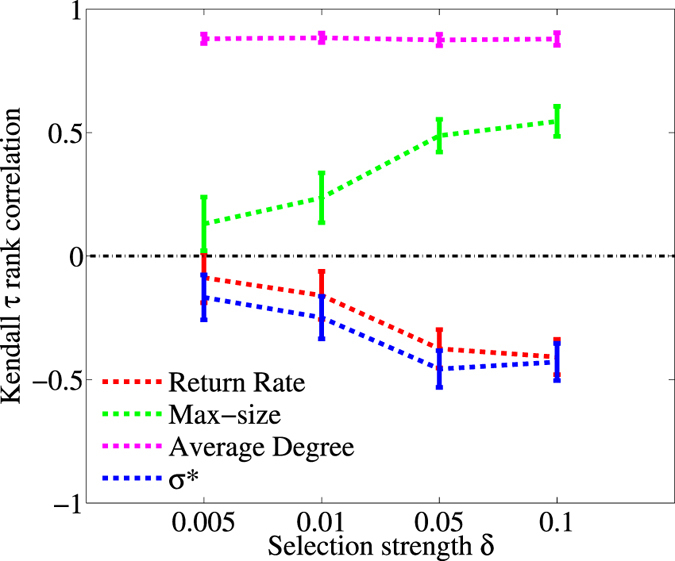
Consistency of the indicators at different selection strength. Mean and standard deviation values of the distributions of Kendall *τ* coefficients for increasing selection strengths *δ*. Non-structural indicators are return rate and max-size; the structural indicators are the structural coefficient σ^*^ and the average degree. Average degree appears to be the strongest indicator. In general, the strength of these indicators is enhanced as the selection increases.

Trends in the indicators are useful if they are consistently correlated to changes in the embedding parameter *q* independently from the strength of selection *δ*. We test for such consistency by computing indicator trends up to the value of *q* where cooperation persistence drops below 0.5. We first generate sequences of indicator values by randomly selecting a value from any of the perturbations at each *q*. Second, we compute the correlation (quantified by Kendall *τ* rank coefficient) between the constructed indicator sequences and the values of *q* (see Methods for details). By repeatedly performing the above two steps, we derive distributions of Kendall *τ*s across different selection strengths *δ*. We found that the means of the indicators trends are generally consistent: mean values are either positive (increasing trend) or negative (decreasing trend) across different selection strengths (Fig. 5). In particular, trends becomes stronger when selection strength *δ* increases for all indicators except for average degree. Average degree is the most consistent and strongest indicator regardless of whether the selection strength is weak or strong (Fig. 5).

**Figure 6:**
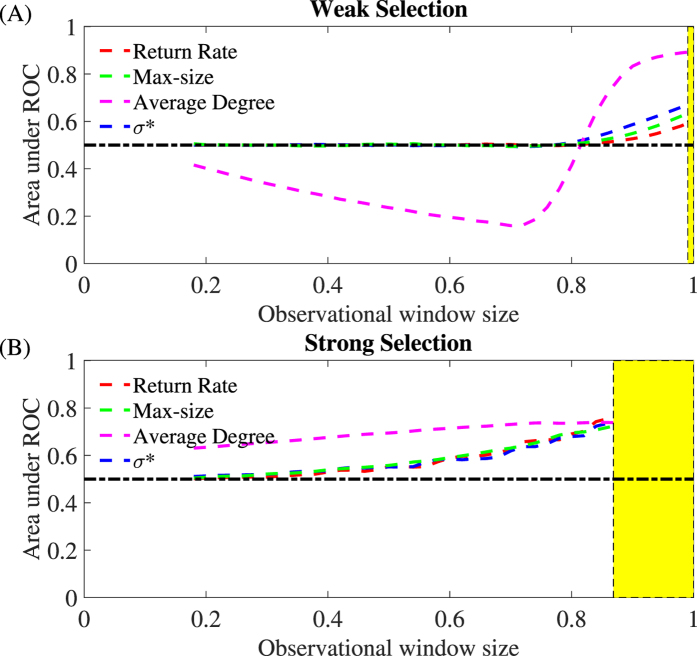
Accuracy in the early detection of the indicators. We plot the area under the receiver operator characteristic (ROC) curves (AUC) for structural and non-structural indicators for weak and strong selections (*δ* = 0.005 and 0.1) and increasing size of the observational window. The size of the observational window is inversely related to the distance from the defined point of cooperation collapse (yellow shaded area). Non-structural indicators are return rate and max-size, and the structural indicators are structural coefficient σ^*^ and average degree. An AUC of 1 represents a perfect test of a rising risk of cooperation loss; an area below 0.5 represents a worthless test. When the selection strength is weak, the average degree displays misleading prediction information as the window size increases. In this scenario, the structural coefficient σ^*^ is generally the most accurate indicator. In general, for strong selection, all indicators are more accurate than for weak selection.

We also analyze how “early” an indicator accurately signals the increasing risk of cooperation loss. In other words, how far from the collapse changes in the indicators significantly signal the risk of collapse. We do this by using receiver operator characteristic (ROC) curves^32^ that estimate true positive and false positive rates for all possible cut-off levels of the indicators (see Methods for details). The larger the area under the ROC curve (AUC), the more accurately an indicator identifies the risk of cheater’s invasions. Areas below 0.5 mean that the indicator trend carries no accurate information about the risk of collapse. We compare the estimated area of the ROC curves for each indicator across a range of observational window sizes (Fig. 6). A small observational window size means that an indicator trend is estimated far from the collapse threshold, while a large window means that trends are measured closer to collapse. As expected, closer to the threshold, all indicators accurately indicate the rising risk of collapse, even in the case of weak selection (window size larger than 0.8, Fig. 6A). Notably, although average degree consistently detects the risk of cooperation loss (Fig. 5), it does accurately so only when close to the collapse in the case of weak selection (large windows size, Fig. 6A). The rest of the indicators show a moderate accuracy even far from the collapse.

## Discussion

Recognizing the conditions that favor the spreading of cheating and the collapse of cooperation in a community has been a major goal in the study of complex adaptive systems^1^,^8^,^38^. Here we approach this issue from a different perspective. We look at the dynamics of cheaters, cooperators, and their interactions to infer the risk of cooperation collapse. Specifically, we argue that the collapse of cooperation in our structurally evolving communities may resemble features of a critical transition - an abrupt change in equilibrium state at the crossing of a threshold. Unfortunately, it is difficult to analytically track the nature of this transition in our stochastic individual based simulations. Nonetheless, the loss of cooperation unfolds in a nonlinear (albeit continuous) way, while the difficulty of restoring cooperation in a community of cheaters may indicate the existence of two alternative states driven by the difference in network structure (Fig. 3, and Section 1 in the Supplementary Material). More notably, we find that return rate (i.e., the inverse of the time necessary for a cheater to get expelled from the community) decreases prior to the collapse in our numerical experiments (Fig. 4). Such slow return rates resemble critical slowing down that is a hallmark of the proximity to bifurcations points^19^,^21^. Although the observed slowing down does not prove the existence of a bifurcation in our communities, it clearly marks the transition to a regime in which there is a high chance of cooperation collapse.

Regardless of the mathematical nature of the transition, we could detect it by a handful of non-structural as well as structural indicators (Fig. 4). We show that the maximum size of invaders after the introduction of a single cheater increases as the risk of collapse rises. Intuitively, this indicator reflects the magnitude of a disturbance that the community suffers without collapsing. When it comes to structural changes in network dynamics (Fig. 4 and Figure S7), we show that single metrics, like average degree, or complex metrics, like σ^*^ (the decreasing ratio of beneficial interactions between cooperators, to detrimental ones between cheaters and cooperators), can be used to detect the increasing risk of cheater’s invasions. So far, the few studies on critical transitions in ecological networks assumed fixed topologies and use non-structural indicators to detect species extinctions^31^,^35^. Network metrics, like connectance, assortativity, or clustering, have been used as structural indicators applied on spatial dynamical models where network structure was derived from cross-correlation matrices^39^,^40^. However, in all cases these network matrices were static. Clearly, monitoring structural changes in evolving communities opens up new opportunities for estimating the risk of abrupt transitions.

In fact, there is a variety of structural metrics that could also be tested as potential indicators to detect cooperation loss. Some of these indicators, like for instance network modularity, have been previously related to the resilience of cooperation in distinct network models^41^. In our model, structural indicators were overall correlated as the risk of cooperation collapse increased: average degree, maximum component, and number of links all increased, whereas modularity and fragmentation decreased (Section 7 in the Supplementary Material). There were, however, subtle differences. For example, trends in average degree were independent of the level of selection *δ* (Fig. 5), while modularity and fragmentation showed stronger strength under weak selection (Figure S8). Our findings agree with the suggestion that is it difficult to define a single best indicator^25^. Instead, this will depend on the specifics of the evolutionary model and most probably the best bet will be to evaluate the consistency and accuracy of multiple indicators. Indeed, tests of the consistency and accuracy of the indicator trends (Figs 5 and 6) show the uncertainty in robustly detecting the loss of cooperation in an evolving community, and resonate with earlier studies that report on a variety of factors that can obscure signals (e.g. spatial heterogeneity, non-equilibrium dynamics, measurement error, environmental stochasticity^31^,^42^).

No doubt, the scenario we explored lacks realism as it is specific to dynamics after a single cheater invasion. Under long-term evolution, multiple cheaters could invade multiple times, or cheaters could randomly mutate into cooperators and vice versa. In our model this would translate into individual newcomers selecting a different strategy than the one employed by the role-model, or cooperators randomly mutating to cheaters. In such case, the dynamics would result in an infinite sequence of collapse and recovery of cooperation^14^. We simulated such scenario of long-term evolution with endogenous mutations and analysed the same structural and non-structural indicators by isolating collapse and recovery events (Figure S9). Consistently to the results for single invasions, we found that most of the indicators could still detect the increasing risk of cooperation loss even for a range of different mutation rates (Section 8 in the Supplementary Material).

Despite our work is based on a specific dynamical network model, the approach we develop can be easily tested in other evolutionary models of dynamical structured populations^43^,^44^. In particular, it would be worth evaluating these indicators in other models where the resilience of cooperation is correlated with network parameters such as modularity^41^ and connectivity^45^. Also, it is worth detecting the fragility of cooperation in models where new links are not just based on imitation like in our scenario, but newcomers have the ability to select their partners strategically^15^,^16^, or in models where there exists the possibility for dynamical rewiring^17^.

Overall, our results suggest that it may be possible to evaluate the fragility of cooperation by monitoring interactions and the behaviour of individuals in a community. In practice, however, such information will be difficult to obtain. Identifying cheaters from cooperators, or following their interactions in time seems a daunting task. Still, the difficulty for evaluating the proposed signals may depend on the specific application, the type, and the scale of the community in question. For instance, bacterial communities are emerging as a promising experimental tool to validate hypotheses in ecology and evolution^46^. Testing the proposed indicators may be done with cooperative cells that produce public goods. In such bacterial colonies, in principle one could perform a series of perturbation experiments by introducing cells of cheaters (or invaders) and measuring return rate. Recent papers have showed how structured cellular communities can be used to measure slowing down in deteriorating populations^47^, even in the presence of cheaters exploiting public good producing yeast^48^.

Perhaps the most novel conclusion of this work is that we can identify the progressive loss of cooperation by combining non-structural and structural indicators. Although the results we report might be idiosyncratic to the model assumptions, they still confer a promising pattern. As such our study paves the way for testing and developing similar indicators in a variety of evolving dynamical networks, ranging from biological systems to ecological communities and even socio-economic networks.

## Methods

### Evaluation of the Indicators

We estimate two classes of indicators: structural and non-structural. Non-structural indicators consider only the population composition, while structural indicators consider the structure of the network. We compute structural and non-structural indicators for each perturbation experiment. A perturbation experiment consists of the introduction of a mutant in a network where all agents have the opposite strategy. We compute indicators following the invasion of a single cheater in a network of all cooperators. This is done with the following procedure. A network of all cooperators is updated for 1000 steps to remove transients. We then introduce a cheater newcomer. The system is updated until one of the two outcomes is reached: recovery, the cheater fails to invade; or collapse, the cheater invades and cooperation collapses (Fig. 2). We identify by *t*_0_ the beginning of a perturbation (the addition of the cheater) and with *t*_*end*_ the end of a perturbation (either recovery or collapse) and compute the indicators as follows (regardless of whether a perturbation will lead to recovery – unsuccessful perturbation – or collapse – successful perturbation).

• Non-structural indicators.

– Return rate is 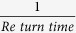, where the *Return time* is the number of update steps the system takes to go back to its original state following a perturbation. Hence, if *t*_0_ is the step at which a perturbation starts and *t*_*end*_ is the step at which the population comes back to its original state, then the return time is defined as *t*_*end*_ − *t*_0_. If the perturbation is successful, then the return rate is defined to be 0.

– Max-size (of cheaters) is the maximal number of cheaters recorded during a perturbation. If the perturbation leads to a complete cheaters invasion then it is the size *N* of the population.

• Structural indicators.

– Structural coefficient σ*:


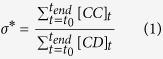


where [*CC*]_*t*_ is the total number of *CC* links (counted twice), [*CD*]_*t*_ is the total number of *CD* links in the network at step *t*. Intuitively σ^*^ evaluates the ratio between the beneficial payoff (generated by purely cooperative interactions) and the detrimental payoff (generated by cheaters connected to cooperators). This coefficient is a simplified version of the structural parameter studied in previous works^43^,^49^.

– Average degree is the average number of links per node recorded during a perturbation.

Figure 4 shows the median of the indicators estimated using 20.000 perturbations for each value of the embedding parameter *q* (increasing from 0 to 1 at an increment of 0.02). We use different selection strengths (*δ* = 0.001, 0.005, 0.01, 0.05), but in the main text we report the indicator results for weak (*δ* = 0.005) and strong selection strength (*δ* = 0.1).

### Consistency of the Indicators - Kendall’s *τ* coefficient

Given two pairs of data, e.g., (*x*_*i*_, *y*_*i*_) and (*x*_*j*_, *y*_*j*_), we say they are *concordant* if *x*_*i*_ > *x*_*j*_ and *y*_*i*_ > *y*_*j*_ or if *x*_*i*_ < *x*_*j*_ and *y*_*i*_ < *y*_*j*_; otherwise if *x*_*i*_ > *x*_*j*_ and *y*_*i*_ < *y*_*j*_ or *x*_*i*_ < *x*_*j*_ and *y*_*i*_ > *y*_*j*_ then we say they are *discordant*. Suppose we have two sequences (corresponding to two variables), each with *n* data points, the Kendall’s *τ* coefficient between the two variables is computed using the number of concordant (cp) and discordant pairs (dp):


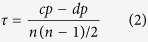


If the agreement between the two variable is perfect the coefficient has a value of 1; if the disagreement is perfect then it has a value of −1; while if the two variables are independent then it is 0. The coefficient can be interpreted as the probability of observing concordant pairs minus the probability of observing discordant pairs^50^.

In our case, for each indicator, we generate a large number of sequences by choosing for each *q* (between *q* = 0 and *q*(0.5)) the value of the indicator obtained in any of the (randomly selected) 20.000 perturbations; *q*(0.5) denotes the value of *q* for which the persistence of cooperation is 0.5. Then, we compute the Kendall’s *τ* coefficient between each of these sequences and the sequence of corresponding increasing values of *q* (again, from *q* = 0 to *q*(0.5)). The obtained distribution of Kendall’s *τ* coefficients is then fitted as a Gaussian one (shown in Figure S4 in the Supplementary Material). Mean values and standard deviation of the distribution are shown in Fig. 5, for all the indicators and various selection strengths.

### Accuracy of the Indicators - ROC curves

In Fig. 6, for each indicator and each value *q*′ of the observational window size, we plot the area under the ROC curve (AUC) computed using only the data between *q* = 0 and *q*′. Such ROC curve is computed in the following manner.

We first define true/false positive/negatives as follows. Given a cut-off *c* and two arbitrary points *q*_1_, *q*_2_ with *q*_1_ < *q*_2_, we denote the values of the indicator at *q*_1_ and *q*_2_ by *s*_1_ and *s*_2_, respectively (Fig. 4). We denote by *f*(*q*_1_) and *f*(*q*_2_) the values of the cooperation persistence at *q*_1_ and *q*_2_, respectively (Fig. 3). We can then define the true/false positive/negative using the following discriminatory condition.

- If (1 + *c*)*s*_1_ < *s*_2_ and *f*(*q*_1_) ≥ *f*(*q*_2_), then it is a true positive (TP).
- If (1 + *c*)*s*_1_ < *s*_2_ and *f*(*q*_1_) < *f*(*q*_2_), then it is a false positive (FP).
- If (1 + *c*)*s*_1_ ≥ *s*_2_ and *f*(*q*_1_) < *f*(*q*_2_), then it is a true negative (TN).
- If (1 + *c*)*s*_1_ ≥ *s*_2_ and *f*(*q*_1_) ≥ *f*(*q*_2_), then it is a false negative (FN).

Considering a large number of randomly formed pairs (*q*_1_, *q*_2_), with 0 ≤ *q*_1_ < *q*_2_ ≤ *q*′ we compute the total number TP, FP, TN and FN. Once these values have been obtained, we can compute the sensitivity and specificity for the indicator as:


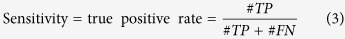



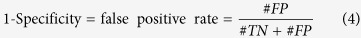


The calculated pair (sensitivity, 1-specificity) constitutes a single point of the ROC curve. Repeating the described process for a large range of cut-offs (from very small to very large), we obtain a set of pairs (sensitivity, 1-specificity) that constitutes a full ROC curve (shown in the Supplementary Material, Figure S5) from which the AUC is then obtained.

Clearly, each indicator has two possible symmetric ROC curves, depending whether the discriminatory condition used to determine FP, FN, TP, TN is either the one presented above or the dual one. For convention, we choose the one that gives the largest AUC when computed with the the largest observational window (i.e., when *q*′ = *q*(0.5), i.e., the *q* for which persistence of cooperation is 0.5), and use that condition to compute the ROC for all other observational windows (i.e., for all other *q*′ < *q*(0.5)).

## Additional Information

**How to cite this article**: Cavaliere, M. *et al*. Detecting the Collapse of Cooperation in Evolving Networks. *Sci. Rep.* **6**, 30845; doi: 10.1038/srep30845 (2016).

## Supplementary Material

Supplementary Information
