# Supplementary material for: Detecting the Collapse of Cooperation in Evolving Networks
Source: Sci Rep. 2016 Aug 5;6:30845. doi: 10.1038/srep30845 (PMC4974622; doi:10.1038/srep30845)
Supplement: Supplementary Information [file srep30845-s1.pdf]

Supplementary Materials

Detecting the Collapse of Cooperation in Evolving Networks

Matteo Cavaliere, Guoli Yang, Vincent Danos, Vasilis Dakos

1 Restoration of Cooperation: How many cooperators are necessary?

As we have already discussed in the main text, it is generally difficult to restore cooperation once it has been lost. Most addition of a cooperator mutant in a network of all cheaters are unsuccessful (Figure 3B in the main text). However, cooperation can be easier restored when cooperators succeed to reach a certain fraction of the population (Figure S1). The restoration of cooperation here is conditional on the fraction of mutants, and it is computed by $\psi_\rho = \frac{\#transitions}{\#perturbations_\rho}$, where $\#transitions$ is the number of transitions from all D to all C and $\#perturbations_\rho$ is the number of perturbations reaching $[C]/N = \rho$. Even in this scenario, however, there is no abrupt transition that can be observed for the restoration of cooperation.

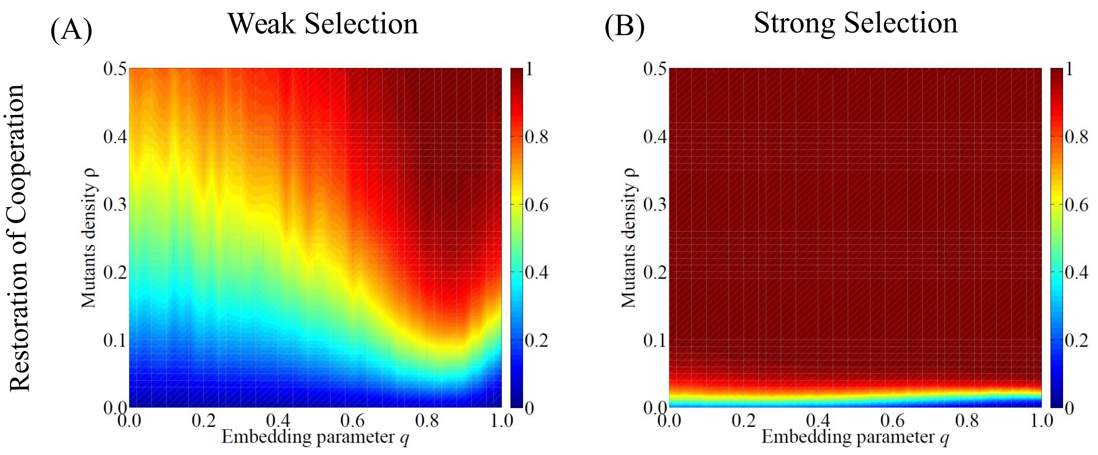

Figure S1. Restoration of cooperation for different densities of mutants. Restoration of cooperation as a function of the embedding parameter q and the densities of mutants (cooperators) at weak ($\delta = 0.005$) and strong selection ($\delta = 0.1$) respectively. Restoration of cooperation is the fraction of successful perturbations in a population of all cheaters, computed by considering 20.000 perturbations and evaluating only those ones where cooperators reach the specified proportion on the total population. Following the methodology explained in the main text, each perturbation is done by updating a network for a sufficiently long time followed by the addition of a mutant cooperator.

2 Effects of Embedding Parameter p

Results in the main text have been obtained by changing the embedding parameter q and fixing the embedding parameter $p = 0.6$. However, the effect of the embedding parameter p is minimal on the persistence of cooperation. In Figure S2, we plot the persistence of cooperation as a function of the embedding parameters p and q at weak ($\delta = 0.005$) and strong ($\delta = 0.1$) selection.

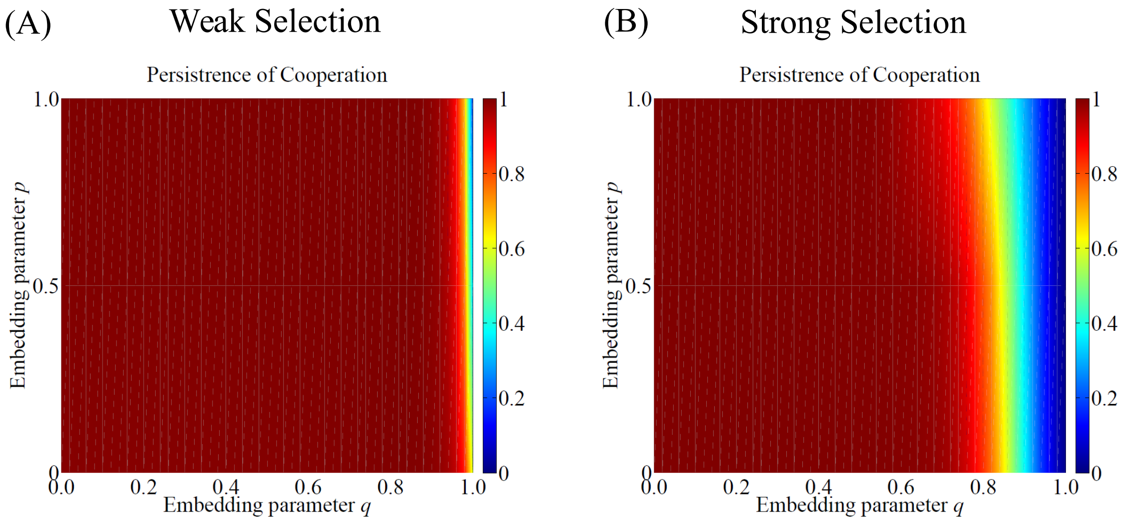

Figure S2. Persistence of cooperation. The probability of cooperation persistence is presented as a function of the embedding parameters q and p for various selection strengths δ. The persistence of cooperation is computed as $1 - \psi$, where ψ is the fraction of successful perturbations, computed by considering 20.000 perturbations. As in the main text, each perturbation is done by updating a network for a long time followed by the addition of a mutant cheater.

3 Different Selection Strengths

In the main text, we have analysed the trends of the indicators using two types of selection strengths: weak ($\delta = 0.005$) and strong ($\delta = 0.1$). Here, we will analyse the indicators for other intermediate selection strengths ($\delta = 0.01$ and $\delta = 0.05$) in Figure S3. In particular, we present the indicators of return rate, max-size, average connectivity, and structural coefficient σ^* following the methodology presented in the main text. We can observe that the qualitative trends discussed in the main text remain unchanged for different selections.

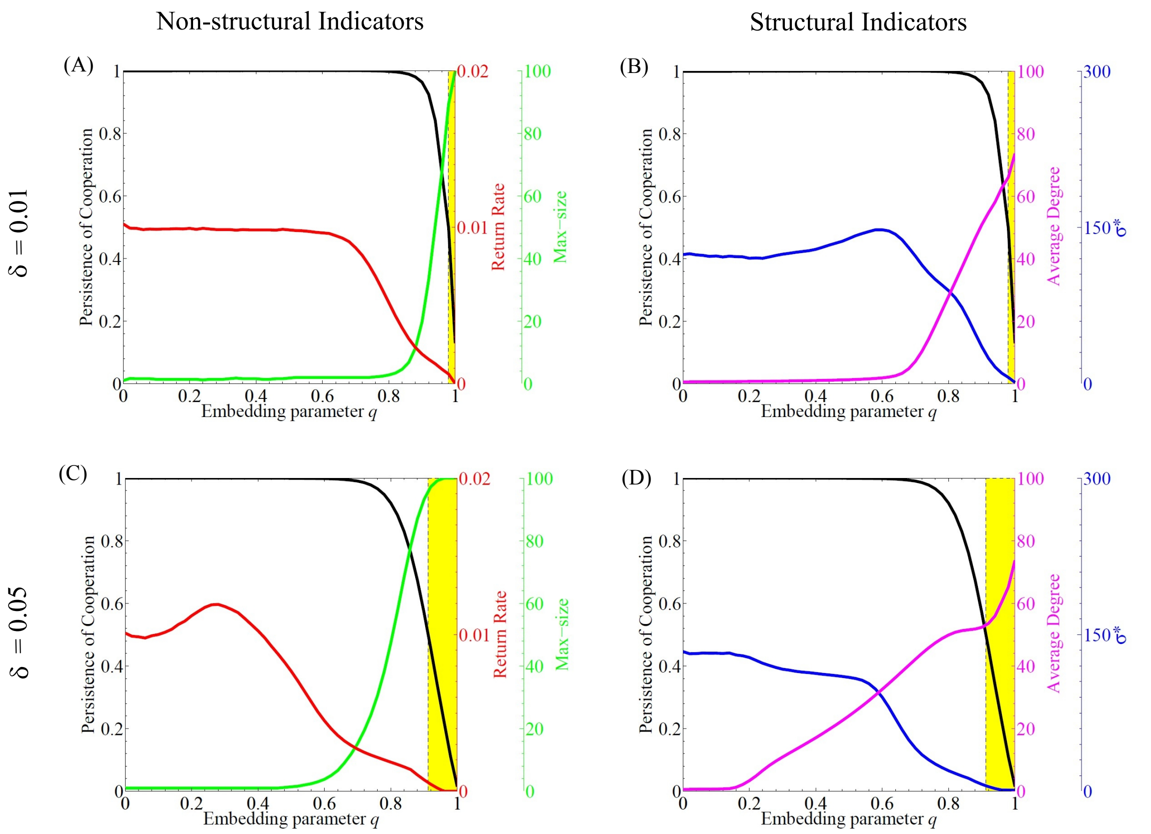

Figure S3. Detecting the loss of cooperation. Structural and non-structural indicators for detecting the loss of cooperation after the invasion of a single cheater for increasing levels of embedding parameter q. Non-structural indicators are 1) return rate (the inverse of the time the system takes to go back to its original state following a perturbation), and 2) max-size (the maximal number of cheaters recorded during a perturbation). Structural indicators are 1) structural coefficient σ^* that evaluates the ratio between the purely cooperative interactions and the interconnections connecting cheaters and cooperators, and 2) average degree (the average number of links per node). Upper row: $\delta = 0.01$; lower row: $\delta = 0.05$. The black curves denote the persistence of cooperation. The yellow shaded area identifies the values of q where cooperation persistence falls below 0.5 (our defined threshold for cooperation collapse). Each point in the indicator curve indicates the median value obtained by considering 20.000 perturbation experiments.

4 Consistency of the Indicators: Kendall τ Distributions

In the main text, we have analysed the consistency of the indicators for different selection strengths by illustrating the mean and the standard deviation values of the Kendall τ distributions (Figure 5 in the main text). Here, we present the actual Kendall distributions. Each Kendall τ coefficient is computed between a sequence of (randomly picked) indicators and the linearly increasing curve of qs up to the threshold $q(0.5)$. Using a standard bootstrap approach without replacement we consider a large number

of indicator sequences and the corresponding set of Kendall τs values is then used to fit a Gaussian distribution (Figures S4) (for more details see the Methods in main text).

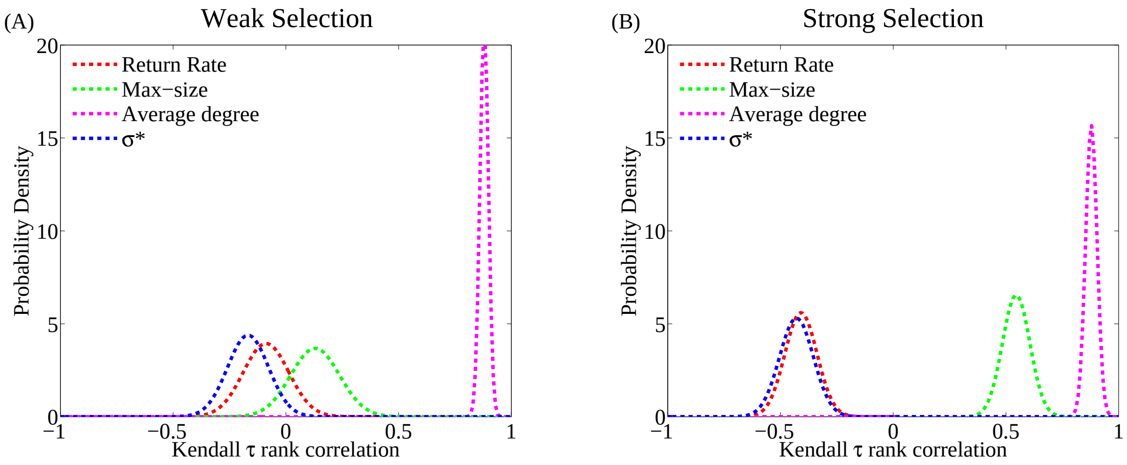

Figure S4. Consistency of the indicators: Kendall τ distributions. Distributions of Kendall τ rank coefficients for structural and non-structural indicators correlated with the increasing embedding parameter q. Here we report $\delta = 0.005$ and $\delta = 0.1$, that are the weak and strong selection considered in the main text. Non-structural indicators are 1) return rate and 2) max-size. Structural indicators are 1) structural coefficient $\sigma*$ and 2) average degree.

5 Receiver Operating Characteristic (ROC)

In main text (Figure 6) we have analysed the accuracy of the indicators by defining the cases of false positives (i.e., false alarms) and false negatives (i.e., missed alarms) for a rising risk of cheaters invasion. This is done by using receiver operating characteristic (ROC) curves obtained by plotting the true positive rates versus the false positive rates for all possible cut-off levels of the indicator values. The larger the area under the ROC curve (AUC), the more accurately an indicator identifies the rising risk of cheater's invasions (areas below 0.5 mean that the indicator trend carries no accurate information about the risk of invasion). In particular, in the main text, Figure 6 shows the areas under the ROC curves for each indicator compared across a range of observational window sizes - the observational window characterizes the distance from the collapse of cooperation (see Methods in the main text for a more detailed description of the ROC curve computation).

For completeness, in Figure S5, we provide the full ROC curves obtained for the largest observational window (i.e., with the data from $q = 0$ to $q(0.5)$).

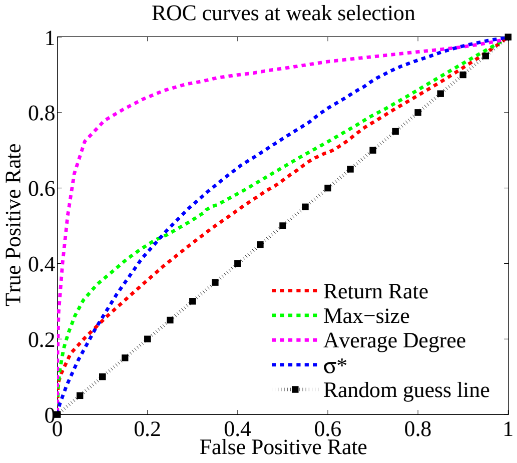

(a) Condition I, Weak Selection

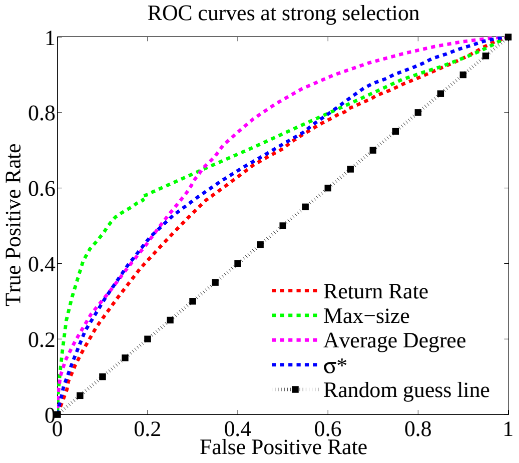

(b) Condition I, Strong Selection

Figure S5. ROC curves. ROC curves obtained for the largest observational window from $q = 0$ to $q(0.5)$, for weak ($\delta = 0.005$) and strong ($\delta = 0.1$) selection.

6 Percentiles of Indicators

In Figure S6, we plot the median of the indicators considered in the main text (Figure 4) as well as the range between 25% percentile and 75% percentiles to show the distribution of the various indicators. Both weak selection $\delta = 0.005$ and strong selection $\delta = 0.1$ are considered.

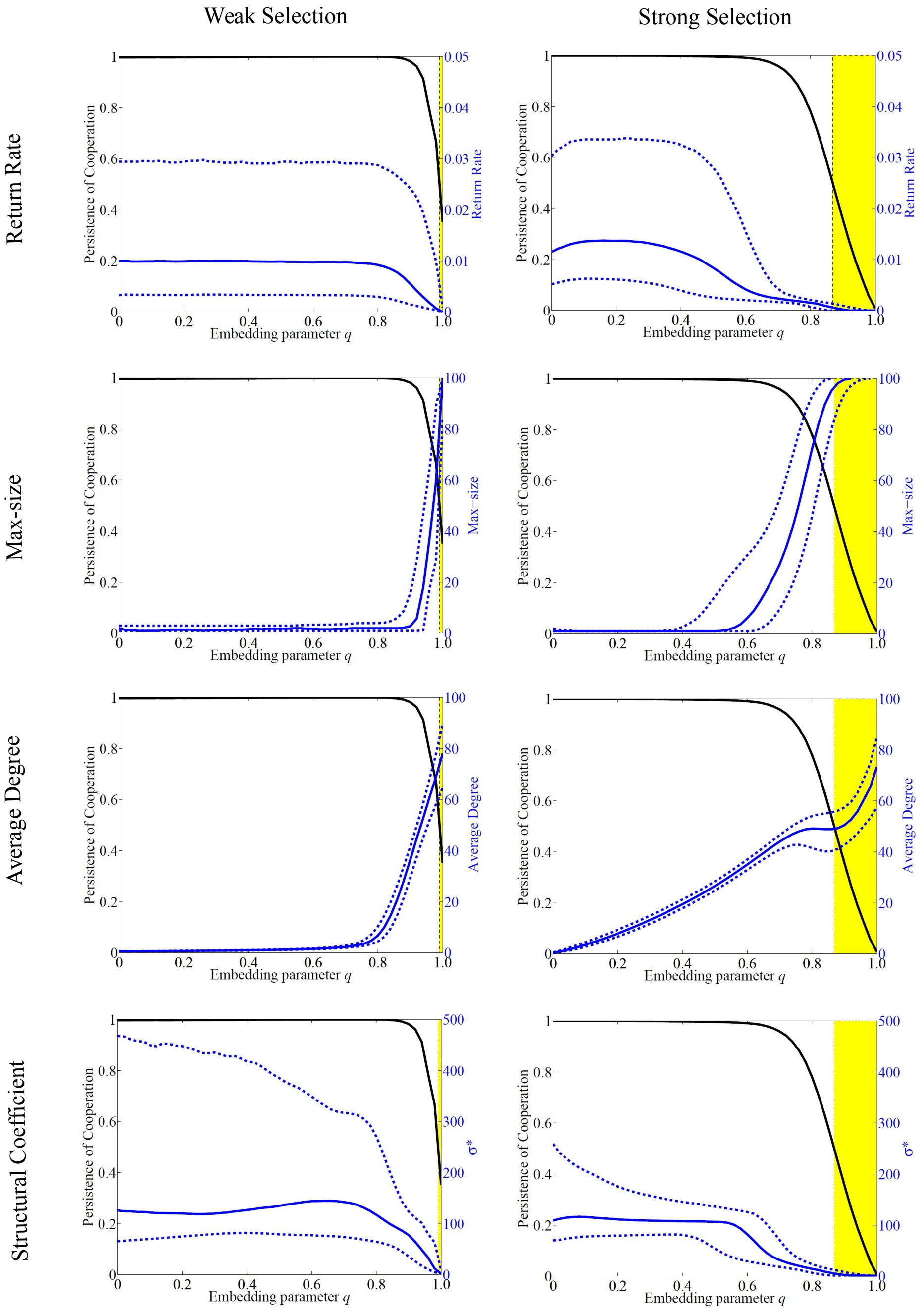

Figure S6. Indicators with 25% percentiles and 75% percentiles. Median values (solid blue curves) of the indicators are obtained from 20.000 perturbations and the dashed blue curves denote the 25% and 75% percentiles. Yellow area starts from the point the persistence of cooperation (black curve) falls below 0.5.

7 More Structural Indicators

In this section, we evaluate other possible structural indicators. In particular, we focus on the number of links between cooperators, between cooperators and cheaters, as well as the size of the largest connected component, fragmentation coefficient and modularity of the network [4].

Following the methodology described in the main text, we evaluate the values of the indicators along a perturbation. Again, we identify by t_0 the beginning of a perturbation and with t_{end} the end of a perturbation.

For each perturbation, we compute these structural indicators in the following manner.

- average number of CC and CD links (in short, [CC], [CD]).

$$[CC] = \frac{\sum_{t=t_0}^{t=t_{end}} [CC]_t}{t_{end} - t_0} \tag{1}$$

$$[CD] = \frac{\sum_{t=t_0}^{t=t_{end}} [CD]_t}{t_{end} - t_0} \tag{2}$$

  where $[CC]_t$ is the total number of CC links (counted twice), $[CD]_t$ is the total number of CD links in the network at step t.

- the size of the largest connected component (in short, max-component).

$$\textit{max-component} = \frac{\sum_{t=t_0}^{t=t_{end}} \textit{max-comp}_t}{t_{end} - t_0} \tag{3}$$

  where $\textit{max-comp}_t$ denotes the size of the largest connected component in the network at step t.

- fragmentation coefficient.

  The network fragmentation coefficient [3, 4] at step t is defined as:

$$\textit{fragmentation}_t = 1 - \sum_{i=1}^{n} \frac{[c_i] \times ([c_i] - 1)}{N \times (N - 1)} \tag{4}$$

  where $c_1, c_2, \cdots c_n$ are the n connected components of the network, with size $[c_1], [c_2], \cdots [c_n]$, respectively.

The fragmentation coefficient in then defined as:

$$fragmentation = \frac{\sum_{t=t_0}^{t=t_{end}} fragmentation_t}{t_{end} - t_0} \quad (5)$$

- modularity measures the strength of the division of a network in modules (i.e., communities). It can be defined as:

$$\sum_i (e_{ii} - a_i^2) \quad (6)$$

where e_{ii} is the fraction of edges in the network that fall within group i and a_i is the fraction of edges that are incident to nodes present in group i. We compute modularity using the algorithm proposed in [5] that allows to detect the community structure and measure modularity.

Following the methodology described in the main text, we evaluate in Figure S7 the persistence of cooperation and the median values of $[CC]$, $[CD]$, max-component, fragmentation and modularity, by considering 20.000 perturbations, at different selection strengths. We also evaluate the consistency of the indicators plotting the mean and standard deviation values of the distributions of Kendall τ coefficients (Figure S8). These results highlights that the strength of an indicator depends on the degree of selection and on the mechanism employed by the indicator.

8 Long-Term Evolution Scenario

In this section, we analyse the scenario where cheaters appear as an endogenous mutation from the role-model (rather than a single introduction, as in the considered perturbations). In this scenario, the dynamics of the system are similar to Figure 1 in the main text with the only difference that the newcomer adopts (with probability μ, mutation rate) a strategy different from the role-model.

For each embedding parameter q, we consider a long simulation composed of $t_{\max} = 10^8$ update steps (we denote by t_0 the beginning of the simulation). We use a mutation rate $\mu = 0.0001$ and start from a random network with $N = 100$ and average degree 4.

The typical dynamics of this system (for three different qs) is shown in Figure S9. When the system is in the state of only Cs, the mutant that appears, can either spread leading the system to the state of only

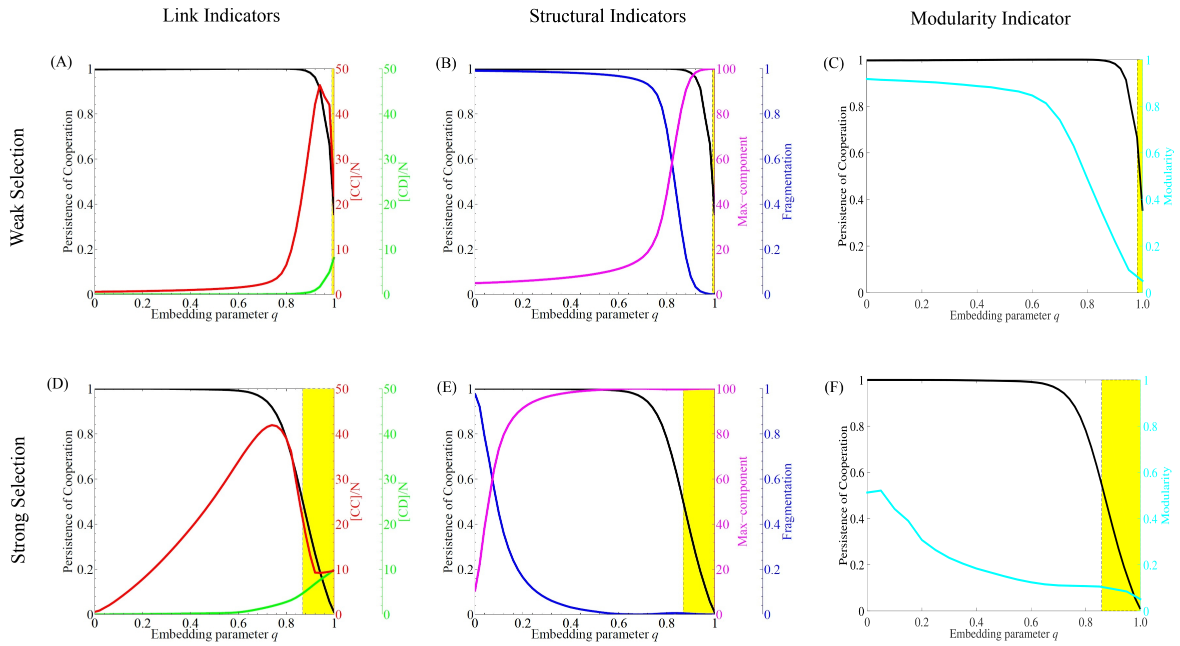

Figure S7. Detecting the loss of cooperation through structural indicators. We plot $[CC]$, $[CD]$, max-component, fragmentation and modularity for weak selection ($\delta = 0.005$) and strong selection ($\delta = 0.1$). The persistence of cooperation is presented in black and the yellow shaded area identifies the values of q where cooperation persistence is below 0.5. The x axis reports the embedding parameter q while $p = 0.6$. Each point in the indicator curve indicates the median value of the corresponding indicator obtained by considering 20.000 perturbations.

Ds (successful invasion) or can be removed from the system before has fully invaded (failed invasion). For failed invasions, a recovery is defined as the number of steps between the addition of a mutant (cheater) in a network of all cooperators and the complete disappearance of cheaters. For a successful invasion, the recovery from the defection regime back to the cooperation regime requires a much longer time, which contains the CD state, DD stage and DC stage shown in Figure S9.

We define the stability of the system based on the number of successful invasions. Figure S10 shows the peak of instability obtained for a large q. After such a peak, the system will more likely stay in the regime of defection. Therefore, in this long-term scenario, we regard such a peak as the threshold of cooperation collapse.

For these runs, we evaluate non-structural and structural indicators as follows:

- Non-structural indicators

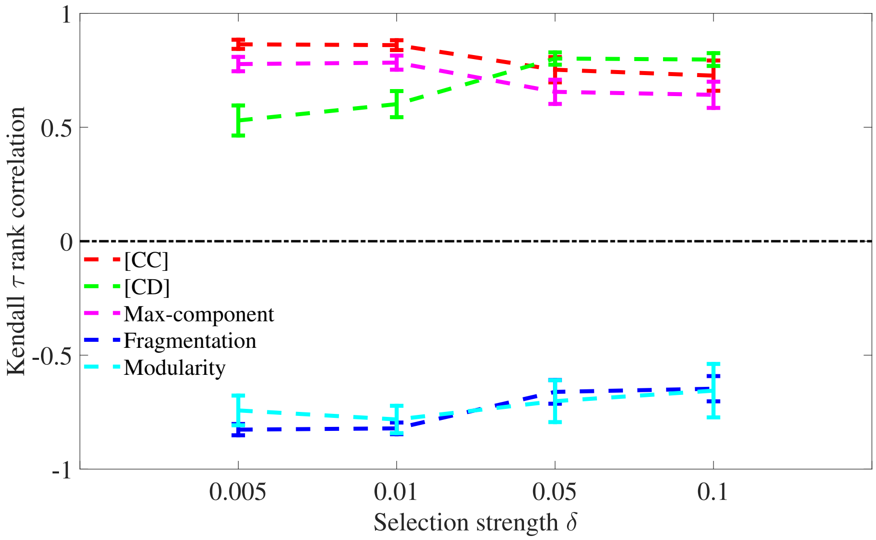

Figure S8. Consistency of the indicators at different selection strengths. Mean and standard deviation values of the distributions of Kendall τ coefficients for increasing selection strengths δ. Some structural indicators are the number of cooperative links [CC], of cooperator-cheater links [CD], the largest component (max-component), network fragmentation and modularity. The strength of indicator depends on the intensity of selection and on the mechanism of the indicator: some of the indicators are stronger when selection strength is weaker while others appear to improve their strength as selection intensity increases.

- Long-term average return rate (*Return rate*) is the mean value of all the return rates obtained by considering all recoveries. If we define by *return time*(i) the return time of the ith observed recovery, then the corresponding return rate is computed as *return rate*$(i) = 1/$*return time*(i). The long-term average return rate is then computed as:

$$Return\ rate = \frac{\sum_{i=1}^{n_r} return\ rate(i)}{n_r} \tag{7}$$

where n_r is the total number of recoveries.

- Long-term average max-size is the average number of maximal amount of cheaters obtained by considering all observed recoveries.

$$Max\text{-}size = \frac{\sum_{i=1}^{n_r} Max\text{-}size(i)}{n_r} \tag{8}$$

where *Max-size*(i) is the maximal number of cheaters recorded in the ith recovery. If the cheater's

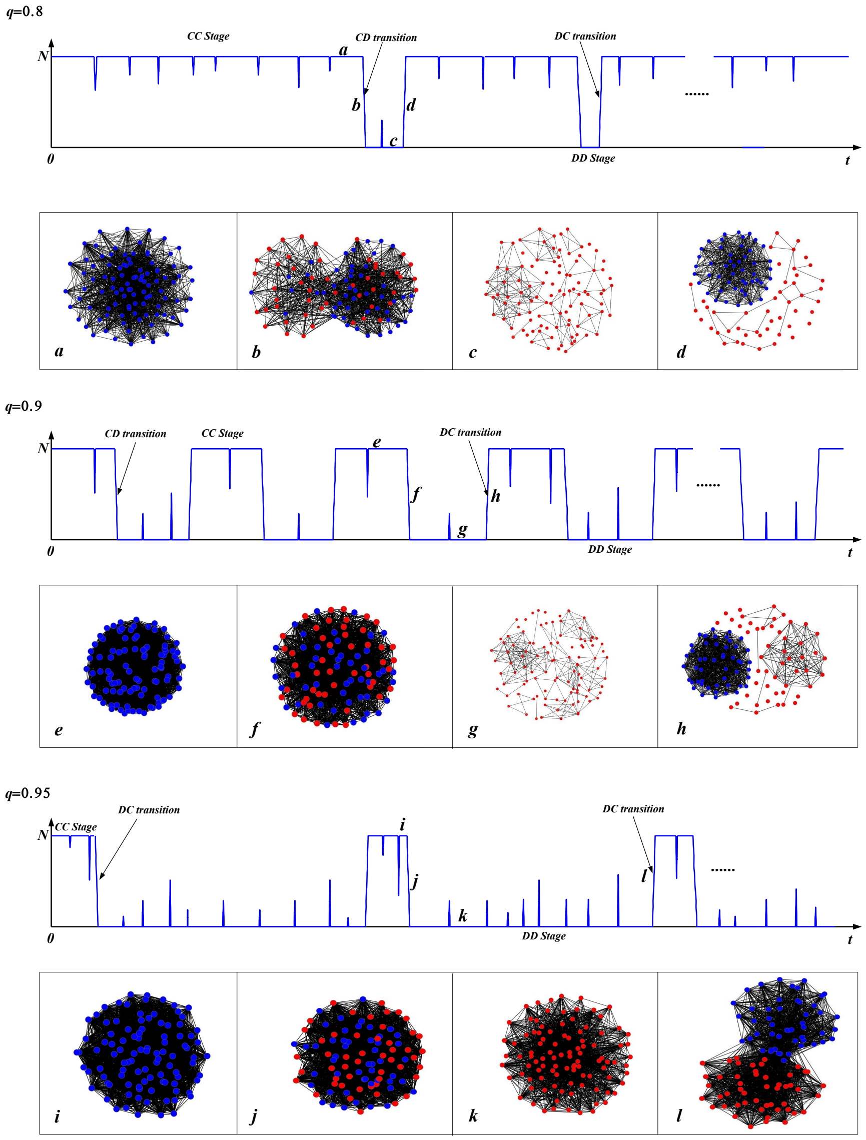

Figure S9. A schematic diagram that shows the evolution of dynamical networks for three different qs. The system starts with a random network with 100 nodes and average degree 4, and all nodes are cooperators. With the mutation rate $\mu = 0.0001$ and selection strength $\delta = 0.01$, we can find the alternation of all-cooperators and all-defection regimes.Specifically, we can identify four different stages: CC (networks a, e and i), CD(networks b, f and j), DD (networks c, g and k) and DC (networks d, h and l). With the increase of q the duration of CC stages is shrinking, while the duration of DD stages is increasing. Notably, when compared versus q we can observe that the number of transitions from the state of only Cs to the state of only Ds follows a non-monotonous curve (Figure S10).

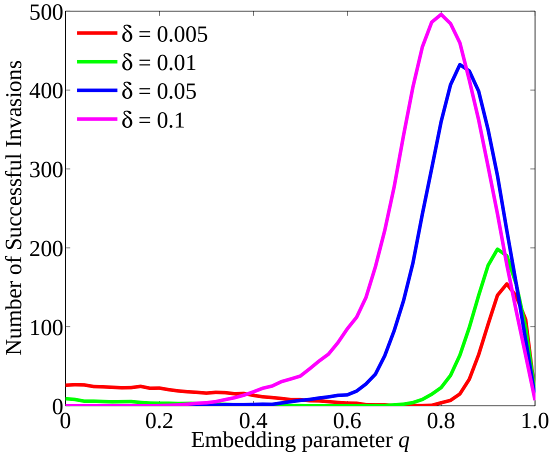

Figure S10. The number of successful invasions in long-term evolution for different selection strengths. We consider a long simulation of 10^8 steps, mutation rate is $\mu = 0.0001$ and we start from a random network with $N = 100$ and average degree 4.

invasion is successful then this amount is exactly N, i.e., the total population size.

- Structural indicators

  - Long-term average degree is the average number of links of each node obtained considering all steps from t_0 to $t_{\max}$:

$$Average\ degree = \frac{\sum_{t=t_0}^{t_{\max}} \langle k \rangle_t}{t_{\max} - t_0} \tag{9}$$

where $\langle k \rangle_t$ is the average degree of the network at step t.

  - Long-term structural coefficient σ^* is obtained by considering all steps from t_0 to $t_{\max}$:

$$\sigma^* = \frac{\sum_{t=t_0}^{t_{\max}} [CC]_t}{\sum_{t=t_0}^{t_{\max}} [CD]_t} \tag{10}$$

where $[CC]_t$ is the total number of CC links (counted twice) and $[CD]_t$ is the total number of CD links in the network at step t.

In Figure S11 we present structural and non-structural indicators (in their defined long-term interpretation) and the number of successful invasions (shown in Figure S10). Figure S11 suggests that almost all indicators discussed in the main text appears to be effective also in the long-term evolution scenario.

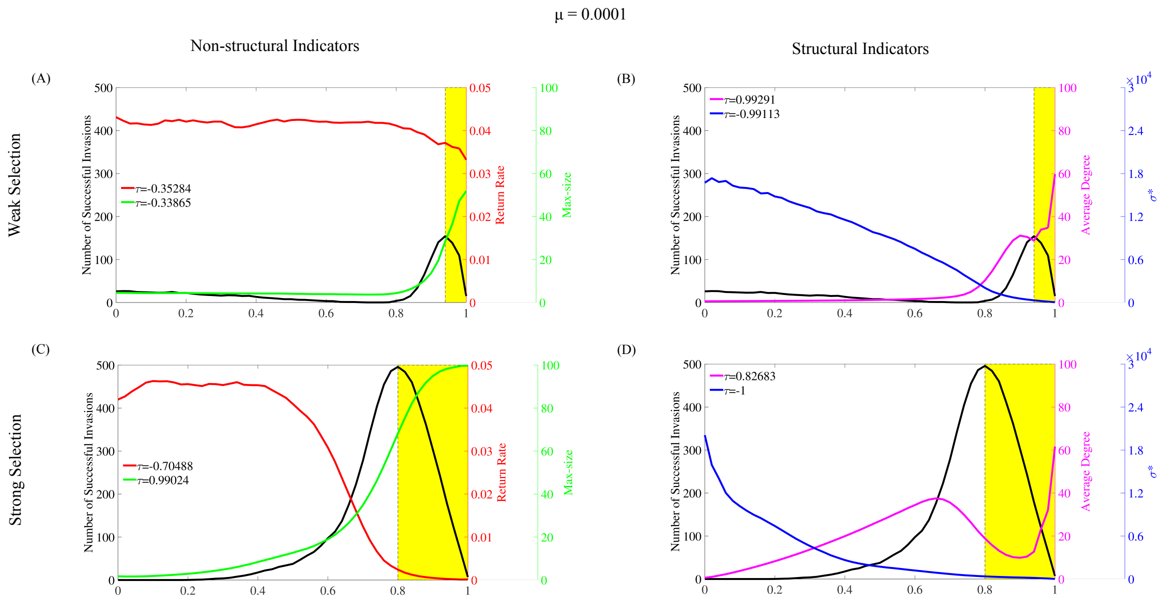

Figure S11. Detecting the loss of cooperation in the long-term evolution. Indicators and the number of successful invasions (black curve) are provided at weak ($\delta = 0.005$) and strong ($\delta = 0.1$) selection. Kendall τ coefficients are computed between the shown curves of indicators and the increasing sequence of q using the data between $q = 0$ and the q corresponding to the collapse of cooperation (i.e., starting of the yellow shaded area). The x axis reports the embedding parameter q while $p = 0.6$.

8.1 Effects of Mutation Rate

We study the effects of mutation rate on the number of invasions and on the success of the indicators. When the mutation rate μ is changed, one can observe a change in the number of successful invasions (Figure S12). In particular, an increase in the mutation rate leads to more instability with more frequent shifts from cooperation to defection. Interestingly, the value of q that corresponds to the maximal amount of transitions (i.e., the starting point of the yellow area, cooperation collapse) decreases for increasing μ.

We also evaluate the non-structural and structural indicators for two different mutation rates. Independently of mutation rate, the analyzed indicators change in distinct ways before cooperation collapse (Figure S13). Hence, the effectiveness (Kendall values shown in Figure S11 and Figure S13) of the indicators is preserved for a wide range of mutation rates.

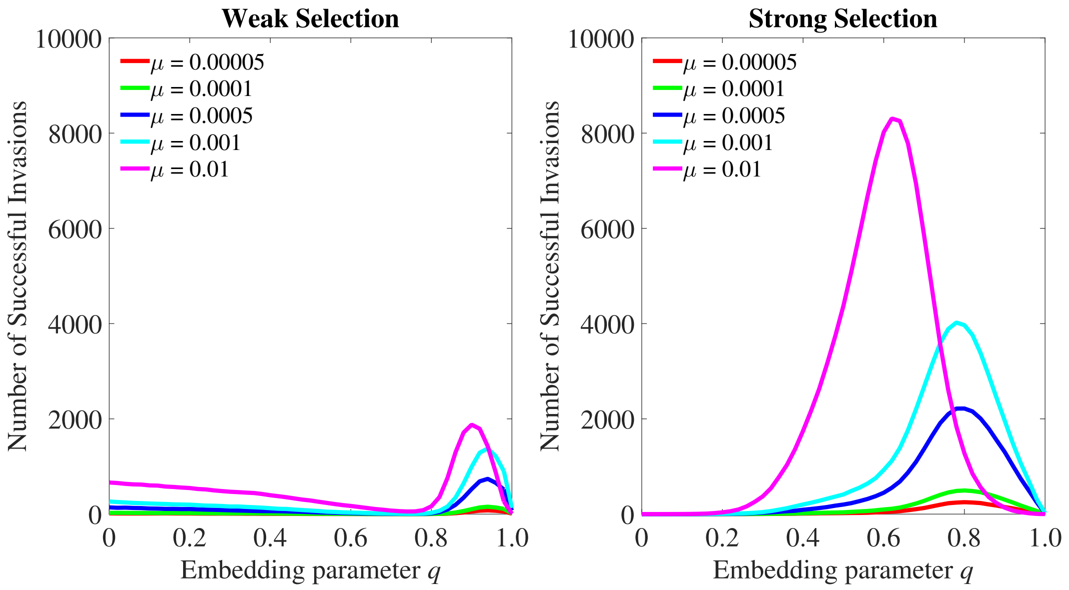

Figure S12. The number of successful invasions for different mutation rates. For weak and strong selection, we consider increasing mutation rates. For each q, we produce a long-term simulation of 10^8 steps, starting with a random network with $N = 100$ and average degree 4. The q corresponding to the maximal number of invasions decreases for increasingly larger mutation rates.

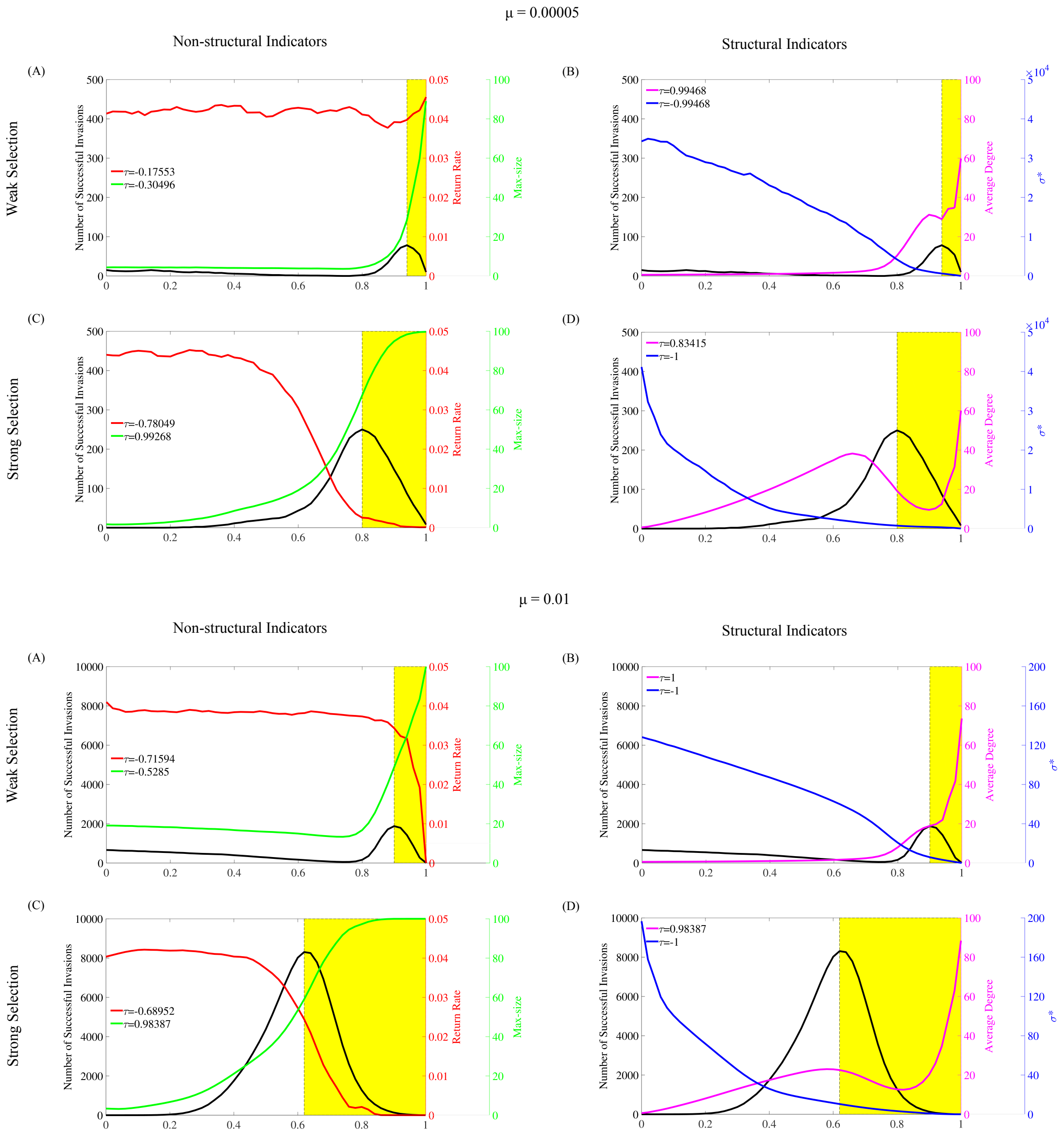

Figure S13. Detecting the loss of cooperation in the long-term evolution. Indicators and the number of successful invasions (black curve) are provided for different mutation rates ($\mu = 0.00005$, above panel, and $\mu = 0.01$, below panel) under weak ($\delta = 0.005$) and strong ($\delta = 0.1$) selection. Kendall τ coefficients are computed between the shown curves of indicators and the increasing sequence of q using the data between $q = 0$ and the q corresponding to the collapse of cooperation (i.e., starting of the yellow shaded area). The x axis reports the embedding parameter q while $p = 0.6$.

References

1. C.E. Tarnita et al. Strategy selection in structured populations. *Journal of Theoretical Biology*, 259, 3, 2009.

2. M.A. Nowak, C.E.Tarnita,T. Antal. Evolutionary dynamics in structured populations. *Philosophical Transactions of the Royal Society B*, 365, 2010.

3. Y. Chen et al. Percolation theory and fragmentation measures in social networks *Physica A: Statistical Mechanics and its Applications*, 378, 1, 2007.

4. S.P. Borgatti. Identifying sets of key players in social networks. *Computational & Mathematical Organization Theory*, 12, 1, 2006.

5. M.EJ. Newman. Fast algorithm for detecting community structure in networks. *Physical review E*, 69, 6, 2004.
